# Treatment of Diabetes and Osteoporosis—A Reciprocal Risk?

**DOI:** 10.3390/biomedicines10092191

**Published:** 2022-09-05

**Authors:** Agnieszka Zawada, Alicja Ewa Ratajczak, Anna Maria Rychter, Aleksandra Szymczak-Tomczak, Agnieszka Dobrowolska, Iwona Krela-Kaźmierczak

**Affiliations:** 1 Department of Gastroenterology, Dietetics and Internal Diseases, Poznan University of Medical Sciences, 61-701 Poznn, Poland; 2 Doctoral School, Poznan University of Medical Sciences, 61-701 Poznan, Poland

**Keywords:** antihyperglycemic agents, diabetes, osteoporosis, diet

## Abstract

Diabetes mellitus is a metabolic and systematic disorder that requires individualized therapy. The disease leads to various consequences, resulting in the destruction of tissues and organs. The aforementioned outcomes also include bone mineral disorders, caused by medications as well as diet therapy and physical activity. Some drugs may have a beneficial effect on both bone mineral density and the risk of fractures. Nevertheless, the impact of other medications remains unknown. Focusing on pharmacotherapy in diabetes may prevent bone mineral disorders and influence both the treatment and quality of life in patients suffering from diabetes mellitus. On the other hand, anti-osteoporosis drugs, such as antiresorptive or anabolic drugs, as well as drugs with a mixed mechanism of action, may affect carbohydrate metabolism, particularly in patients with diabetes. Therefore, the treatment of diabetes as well as osteoporosis prevention are vital for this group of patients.

## 1. Introduction

According to the definition, diabetes mellitus (DM) represents a group of metabolic disorders characterized by chronic hyperglycemia, which stems from impaired production or action of insulin. The incidence of osteoporosis in patients with diabetes is influenced by a number of factors, such as the co-occurrence of obesity, the type and duration of diabetes, the presence of chronic complications, as well as the type of therapy applied. In type 2 diabetes, increased body fat inhibits the expression of genes involved in osteoblastogenesis. Furthermore, it also directly increases the expression of peroxisome proliferator activated receptor gamma (PPARγ receptors) and stimulates the Wnt/protein kinase C pathway [1,2]. Additionally, hyperglycemia occurring in diabetes induces oxidative stress and the formation of reactive oxygen species and advanced glycation end products-AGE (protein glycation) [3]. This, in turn, leads to the inhibition of osteoblasts, stimulation of osteoclasts, and increased bone turnover [4]. In type 1 diabetes, increased expression of growth factors IGF-1 and TGF-B1 results in a decreased bone mineral density. Conversely, in type 2 diabetes, excessive hyperinsulinemia leads to the stimulation of the IGF-1 receptor present on osteoblasts, resulting in an increased BMD. Nevertheless patients with this type of diabetes are also prone to fractures. Moreover, as experimental studies demonstrate, chronic hyperglycemia also affects every stage of bone formation, e.g., changes in bone microcirculation are observed in patients with a long history of hyperglycemia. In fact, since chronic hyperglycemia impairs osteoblastic function, it also possibly increases bone resorption [5].

Chronic hyperglycemia constitutes a strain on the body and affects various organs and systems [6]. Nevertheless, effective treatment maintains normal glycemia and may prevent the development of chronic consequences of DM [7]. A conceptual breakthrough in diabetes treatment has been visible in recent years. New medications and clinical trials taking into account evidence-based medicine, such as LIDER and EMPAREG OUTCAME, have changed the goal of diabetes treatment to achieve a reduction in glycemia and a decrease in mortality [8,9]. However, the treatment applying new anti-hyperglycemic agents should be supported by behavioral treatment on every step of diabetes therapy, because proper diet and physical activity may also prevent the consequences of diabetes [10]. Bone mineral disorder has been known for a number of years, although it has been underestimated as a complication of DM [11]. Therefore, it is interesting to observe how new, although known for a number of years, drugs together with behavioral treatment affect bone mineral balance. Additionally, diet and supplementation remain underestimated in preventing the abovementioned consequences in patients with DM. Hence, developing guidelines, early diagnosis, and proper supplementation in the early stages of bone mineral disorders may be effective in therapy of this complication among patients suffering from DM.

## 2. Dietary Management of Diabetes and Bone Metabolism: Do They Carry the Risk of Developing Osteoporosis?

### 2.1. Calcium

Calcium intake may protect against overweight and obesity, which are one of the risk factors associated with type 2 diabetes mellitus. Varenna et al. demonstrated an inverse relationship between BMI and increasing the dairy intake [12]. Their study suggests that calcium intake may protect against overweight and obesity as well as osteoporosis. Moreover, dairy calcium intake increases weight loss among patients suffering from T2DM [13], and the consumption of dairy products may help to maintain weight loss [14]. Nevertheless, 25-week long supplementation of calcium did not increase body weight and fat mass loss when compared with placebo [15].

Energy restriction reduces calcium absorption in comparison with a group with normal energy intake. Therefore, calcium intake should be greater during a weight loss diet since normal intake may disturb calcium balance [16]. It is worth bearing in mind that there was a difference in calcium intake in overweight patients who were on the intermittent fasting, Paleolithic, or Mediterranean diets, and the intake was found to be highest among patients on the Mediterranean diet. Additionally, energy intake was the highest in patients on the Mediterranean diet [17].

### 2.2. Vitamin D

Hemoglobin A1c was found to be inversely correlated to vitamin D level among patients suffering from T2DM [18]. According to Nam et al., children with T1DM presented lower concentrations of serum 25OHD and 1,25(OH)_2_ D when compared with the healthy population [19].

Due to the high affinity of adipose tissue for vitamin D, the incidence of obesity promotes the sequestration of vitamin D in adipose tissue. This results in a significant reduction in its serum levels compared to individuals presenting with normal weight [20].

In turn, the excess adipose tissue found in obesity and diabetes promotes the formation of reactive oxygen species, which significantly reduces the bioavailability of vitamin D. In fact, vitamin D decreases the expression of L-type Ca^2+^ channels, which reduces intracellular Ca^2+^ concentration. This, in turn, directly impacts intracellular calcium signaling, affecting insulin secretion by the pancreatic beta cells [21].

By means of affecting the passage of calcium across cell membranes, vitamin D also affects tissue sensitivity to insulin [22]. In addition, it also affects the expression of insulin receptors in the peripheral cells. Modulating the expression and the activity of cytokines reduces the overall pool of inflammatory response in the body, which is particularly important in patients with obesity and diabetes [23,24].

Vitamin D synthesis and metabolism may also be impaired in hepatic steatosis coexisting with obesity and overweight [25]. In addition, high levels of leptin and IL-6 impair the synthesis of this vitamin.

Additionally, the study demonstrated that the risk of abdominal obesity decreased by 8% when serum vitamin D levels increased by 25 nmol/L [26]. Moreover, low levels of vitamin D were also associated with an increase in adiposity and a higher fat infiltration in the muscle tissue [27]. In fact, vitamin D levels were lower among adolescents with obesity when compared with non-obese individuals [28]. It is important to note that low vitamin D levels in children have been linked to obesity but not to other cardiovascular risk factors such as hyperglycemia [29]. Additionally, vitamin D was negatively associated with BMI [30], although no association was found between vitamin D intake and low muscle mass [31]. As the mentioned studies indicate, vitamin D metabolism is significantly related to adipose tissue content. Thus, maintaining its normal levels in metabolic diseases may be crucial for reducing overall inflammation, improving, and compensating for carbohydrate disorders.

### 2.3. Low-Calorie Diet and Bodyweight Reduction

Undoubtedly, type 2 diabetes is strongly associated with obesity. However, patients with type 1 diabetes are more frequently diagnosed with excessive body weight, which is surprising, since clinical features of T1DM have been generally associated with weight-loss [32,33]. Regardless of diabetes type, a low-calorie diet (LCD) must be implemented in every patient with excessive body weight, not only for improving the glycemic control but also for the prevention of exacerbating comorbidities, which are already likely to be at an increased risk due to long-term hyperglycemia. Moreover, LCD should be introduced by a professional dietitian who should guide the patient through every stage of the weight-loss process. It is especially important because a poorly balanced LCD may lead to nutritional deficiencies, which in turn may deteriorate the course of the disease and the resulting comorbidities, including osteoporosis [34,35,36]. However, it should be noted that only a few studies investigated micronutrient deficiencies and LCD, especially in the context of diabetes and osteoporosis, and several individual factors may affect the real-life pattern. Damms-Machado et al. discovered that individuals with obesity undergoing LCD, the lowest micronutrients intake (compared to the dietary reference intake) was observed for retinol, vitamin C, vitamin E, vitamin D, folate, iodine, iron, calcium, and β-carotene—thus, nutrients vital for proper bone health were deficient in the majority of the study group [37]. Gardner et al. assessed micronutrient intake in four weight-loss diets (Atkins, LEARN, Zone, and Ornish) and found that each of them was characterized by inadequate intake of several micronutrients, among them vitamin C, vitamin E, and folate, which could negatively impact bone mineral density in the future [38]. Moreover, alternative weight loss strategies (e.g., very poor ketogenic diet) are becoming increasingly popular among patients and, as with any highly restrictive diet, may increase the risk of deficiencies to an even greater extent (if introduced improperly and without professional care) [39].

## 3. Antidiabetic Drugs and Bone Tissue Metabolism

Antidiabetic drugs may affect bone metabolism in a neutral, positive, or negative way. However, many clinical studies do not correspond to experimental studies.

### 3.1. Metformin

Metformin presents a pleiotropic mechanism, although it may also affect bone metabolism. The main action of metformin includes the activation of AMP-activated protein kinase (AMPK). AMPK may directly influence bone turnover by increasing osteoblastogenesis and decreasing osteoclastogenesis. Metformin increases osteogenesis by activating the AMPK and fructose 1,6-biphosphate pathways [40]. According to the experimental studies, metformin activates the differentiation of mesenchymal stem cells towards osteoblasts and inhibits osteoclasts differentiation [41]. In fact, clinical studies confirm experimental studies. According to Hidayat et al., fractures in patients treated with metformin were reduced, and Salari-Moghaddam et al. also supported the beneficial effects of metformin on bone metabolism [42,43]. Additionally, metformin inhibits adipocyte genesis in the marrow through decreased endothelial nitric oxide synthase (eNOS) [44].

Furthermore, metformin-mediated glucose utilization occurs via protein kinase-dependent on calcium [45]. One of the most common side effects is diarrhea, which may disturb the proper absorption of minerals essential for bone mineralization. However, diarrhea is usually short-term, subsides after a few days following the introduction of treatment, and does not eliminate the long-term positive effect of metformin on bone metabolism. In addition, metformin decreases the negative effects of rosiglitazone on the bone [46].

### 3.2. Sulfonylureas

According to the studies, glimepiride, belonging to sulfonylureas, presents a beneficial effect on the stimulation of bone formation [47]. In fact, glimepiride also inhibits bone loss linked to the menopause [48], although, generally, sulfonylureas are considered neutral for bone metabolism. However, it is vital to notice that sulfonylureas often cause hypoglycemia, particularly among the elderly, leading to an increased risk of fracture [49], which has been confirmed in a study conducted by Starup-Linde et al. They reported that treatment with sulfonylureas in the last 90 days among 5244 patients with DM2 was associated with a higher risk of femoral neck fractures [50].

### 3.3. Insulin Therapy

Similar to sulfonylureas, the clinical impact of insulin on bone stems mainly from the occurrence of hypoglycemia and is related to its risk of falls and bone fractures [51]. The last study, involving 58 853 newly-diagnosed diabetes patients, showed that patients treated with insulin presented a higher risk of osteoporotic fracture amounting to 38% [52]. Other clinical trials presented a higher risk of fractures among patients with DM2, particularly in postmenopausal women [53,54]. Nevertheless, it is currently difficult to account for due to the anabolic impact of insulin.

### 3.4. Thiazolidinoidiones

Thiazolidinoidiones decrease BMD and increase the risk of fractures by eliminating the inhibitory impact of PPAR-γ on the differentiation of osteoclasts, increasing sclerostin production in osteocytes, and increasing adipocyte infiltration in the marrow [55], which was considered for rosiglitazone and pioglitazone [56,57]. Rosiglitazone decreased bone regeneration, influenced trabecular bone in the metaphysis of the femur and decreased the density of osteoblasts and osteoclasts [46]. Additionally, it also affected TRAP-linked epiphyseal growth plate, leading to a decreased bone mass and an increased risk of fractures. The ex vivo study demonstrated that osteogenic involvement had been reduced during treatment with rosiglitazone, leading to an increased expression of PPARγ and decreased Runx2/Cbfa1, inhibiting phosphorylation AMP kinase, and inhibiting osteoblast mineralization and differentiation [46].

### 3.5. GLP-1 Antagonists

It is vital to note the impact of GLP-1 antagonists on bone metabolism and the risk of fractures. Treatment with GLP-1 antagonist decreases bone resorption by affecting osteoclast and osteoblast balance. In vivo studies showed that agonists of the GLP-1 receptor may affect the fat-bone axis through the promotion of osteogenic differentiation and inhibition of adipogenic mesenchymal precursor cells in the bone. Additionally, signaling of Wnt/β-catenin also takes part in this process. Mature osteocytes with GLP-1 receptor produce sclerostin, which inhibits Wnt/β-catenin signaling through binding with the low-density lipoprotein receptor-related protein 5 and preventing Wnt binding. GLP-1 analogues decrease the expression and concentration of sclerostin. The GLP-1 receptors are expressed in C cells in the thyroid, leading to the release of calcitonin, causing inhibition of bone resorption [58]. The anabolic effect of liraglutide and exenatide on GLP-1 receptors in preosteoblasts and osteocytes prevents bone mass loss linked to weight loss, and causes many fractures [59,60,61]. Additionally, GLP-1 may control bone resorption through interaction with GLP-2 and glucose-dependent insulinotropic polypeptide (GIP) and affects the pathway depending on calcitonin [62].

### 3.6. Dipeptidyl Peptidase-4 Inhibitors (DPP-4 Inhibitors)

In the conducted studies, sitagliptin, a dipeptidyl peptidase-4 inhibitor, caused inhibition of bone resorption among postmenopausal women; however, this result was not confirmed in the meta-analysis [63,64]. Additionally, a Korean study did not reveal the influence of DPP4 inhibitors on bone fracture frequency [65]. According to Qiu et al., increased serum DPP4 levels were associated with a higher risk of fractures among newly diagnosed patients with diabetes mellitus type 2 [66]. The cohort study, involving 340 participants and lasting five years, reported that using a DPP-4 inhibitor was linked with a decreased risk of fractures due to any cause and fracture of the upper limbs in patients suffering from type 2 diabetes mellitus [67].

### 3.7. Sodium-Glucose-2 Cotransporter Inhibitors-2

**Figure 1:**
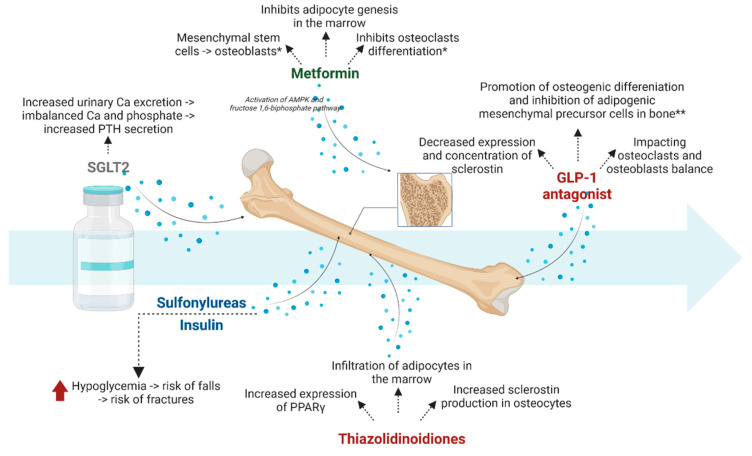
The mechanism of action of drugs used in diabetes on bone mineral metabolism. *—data come from observational studies; **—data come from in vivo studies; Ca-calcium; PPARγ-peroxisome proliferator-activated receptor gamma.

It was speculated that sodium-glucose cotransporter inhibitors, which were regarded by all specialists with a great deal of uncertainty, could affect bone. A hypoglycemic mechanism was associated with increased urinary calcium excretion, which resulted in an imbalance in calcium and phosphate homeostasis (associated with increased PTH secretion, which increases bone resorption) [68]. Similar results were obtained in the CANVAS study, in which a 4% higher incidence of fractures was observed when using canagliflozin [69]. However, these reports were not confirmed in the subsequent studies, such as CANVAS-R and CREDENCE [70,71]. In fact, the association between empagliflozin and bone fractures was also excluded [72]. SGLT2 may also lead to decreased BMD [73], although other studies did not support the effect of these drugs on calcium and phosphorus, vitamin D, or parathyroid hormone (PTH) levels [74]. Therefore, this group of drugs still requires detailed studies regarding their effects on bone. Finally, a meta-analysis by Chai et al. evaluated a total of 177 randomized trials and showed that modern antidiabetic drugs, such as DPP-4i, GLP-1 RA, and SGLT-2, did not increase the overall risk of fractures compared to insulin, metformin or sulfonylureas [75]. Therefore, it would imply that the use of modern and mortality-reducing drugs has no adverse effects on bone metabolism. The mechanism of action of the drugs used in diabetes on bone mineral metabolism has been shown in Figure 1. A summary of the effects of antihyperglycemic drugs on fracture risk has been provided in Table 1.

## 4. Anti-Osteoporosis Drugs and Glucose Metabolism

In the pharmacological treatment of osteoporosis, three groups of drugs are employed—antiresorptive drugs (bisphosphonates, denosumab, and selective estrogen receptor modulators), anabolic drugs (teriparatide), and drugs with a mixed mechanism of action (strontium ranelate).

### 4.1. Antiresorptive Drugs

In terms of antiresorptive drugs, their mechanism of action is mainly based on inhibiting the activity of osteoclasts, whereas anabolic drugs stimulate the action of bone-forming cells [76,77,78]. Bisphosphonates are a basic group of antiresorptive drugs used to treat osteoporosis. Chemically, they are stable inorganic pyrophosphate (PPi) derivatives where two phosphate groups are combined by esterification. Bisphosphonates bind to hydroxyapatite crystals and act through two mechanisms: on the one hand, they inhibit the activity of osteoclasts and stimulate their apoptosis, and on the other they act on osteoclasts [79]. Consequently, bisphosphonates lead to a reduced risk of vertebral fractures and non-vertebral fractures, including the proximal end of the femoral neck [80]. Researchers are also interested in the pleiotropic effects of bisphosphonates—, e.g., anti-cancer effects, modification of lipid concentrations—emphasizing the impact on carbohydrate metabolism. The population study of Toulis et al. showed a 50% reduction in the risk of type 2 diabetes in patients using bisphosphonates [81]. The observations of Karimi Fard et al. also seem to be promising as they showed that the use of oral alendronate at a dose of 70 mg/week for 12 weeks might significantly improve fasting glucose levels. Additionally, it may have a beneficial effect on glycated hemoglobin (HbA1C) in pre-diabetic and postmenopausal women and thus may slow down the rate of progression to diabetes [82]. Bone tissue is a rich source of cytokines, i.e., osteokines, which may affect glucose metabolism. Therefore, it is postulated that antiresorptive drugs modulate bone turnover and act on carbohydrate metabolism. Maugeri D et al. demonstrated a reduction in the daily insulin requirement in patients with type 1 diabetes and osteoporosis who are being treated with alendronate. The authors suggest that the results can be attributed not only to the increase in BMD and slowing down of bone turnover, but above all to the reduction of clinical symptoms of osteoporosis, such as pain and movement limitations, which led to a better physical performance of patients and, therefore, to the improvement of glucose metabolism [83]. Another essential issue in patients with diabetes is renal failure. Hence, it is worth mentioning that special care should be taken in terms of the use of bisphosphonates in this patient group, particularly in individuals with a creatinine clearance of less than 30 mL/min (GFR < 30 mL/min). In fact, it has been shown that intravenous preparations of bisphosphonates can lead to a severe deterioration of GFR, which may be due to the local accumulation of these drugs in the kidneys [84,85]. Nevertheless, no renal complications were observed when oral forms of bisphosphonates were used in patients with mild renal impairment [86]. Denosumab is a fully human monoclonal antibody against the nuclear factor ligand-receptor activator kappaB (RANKL), which blocks its binding to RANK, inhibiting the development and activity of osteoclasts and reducing bone resorption, which in turn leads to an increase in bone mineral density [87]. Denosumab reduces the risk of spinal fractures and non-vertebral fractures, including fractures of the proximal end of the femur [88]. In patients with different stages of renal failure, denosumab has a high efficacy and safety profile, which is clinically relevant [89]. Furthermore, Bonnet et al. showed that RANKL could affect insulin sensitivity and glucose uptake; hence, there have been suggestions that an antibody acting on the RANK/RANKL/OPG system may have an effect on carbohydrate metabolism [90]. However, the Napoli et al. study demonstrated no significant changes in fasting plasma glucose in osteoporotic postmenopausal women with prediabetes or type 2 diabetes following denosumab use [91]. According to the study, no effect was also observed on carbohydrate metabolism parameters in subjects with bone mineral density disorders but without diabetes mellitus [92]. The Passeri et al. study, conducted on patients with severe osteoporosis but without diabetes, also seems promising. The researchers reported that a single dose of 60 mg denosumab did not result in changes in fasting glucose, insulin, or HOMA-IR levels at both 4 and 12 weeks, although it did lead to a significant reduction in liver insulin resistance at 4 weeks and a decrease in HbA1c levels at 12 weeks [93].

Selective estrogen receptor modulators (SERMs) bind to estrogen receptors (ER) α and β and have an agonistic or antagonistic effect depending on the compound itself as well as the target tissue [94]. SERMs reduce the risk of vertebral fractures; however, they do not affect the risk of non-vertebral fractures, including the proximal end of the femoral neck [95]. Tamoxifen has been shown to contribute to numerous metabolic side effects, such as type 2 diabetes, lipid disorders, and fatty liver disease [96]. Hejazi et al. and Sun et al. showed an increased risk of diabetes in patients with breast cancer treated with tamoxifen [97,98]. Furthermore, it is possible that tamoxifen may have adverse effects on the β-cells in the pancreas and impair the survival of pancreatic islets [99]. In fact, treatment with tamoxifen may contribute to decreased insulin sensitivity, although the mechanism has not yet been fully understood [100]. Additionally, the effect of raloxifene on carbohydrate metabolism when used in the treatment of osteoporosis appears to be controversial and may depend on the duration of treatment. In postmenopausal women without diabetes, short-term treatment with raloxifene did not alter fasting blood glucose or insulin levels; however, long-term administration of this medication may contribute to a decreased insulin sensitivity [101,102].

The study by Campos et al. compared the effects of risedronate and conventional osteoporosis treatment based on calcium and vitamin D supplementation in patients with type 1 diabetes mellitus. The study demonstrated an increase in bone formation markers and a significant increase in BMD at the spine and hip [103], and a group of patients with type 1 and type 2 diabetes also showed a beneficial effect of alendronate [104]. In the Fracture Intervention Trial (FIT), alendronate reduced markers of bone turnover (C-terminal telopeptide and bone-specific alkaline phosphatase levels) and improved BMD in women with T2DM) [105]. However, as it has been established, BMD is an insufficient marker of fracture risk, and the vital question is whether an increase in BMD correlates with a reduction in fracture risk. In fact, low bone turnover found in patients with diabetes may be exacerbated via antiresorptive therapy, which may in turn have a detrimental effect on bones.

### 4.2. Anabolic Drugs

Teriparatide is a fragment of 1–34 molecules of human recombinant parathyroid hormone (PTH) and leads to increased bone mineral density by activating bone formation. Teriparatide reduces the risk of cervical fractures and non-vertebral fractures [106]. Conclusions from clinical trials regarding the action of teriparatide on carbohydrate management parameters are often contradictory, and the effect on glucose homeostasis remains unknown, although the role of calcium and its effect on glucose transport to the cell and the regulation of insulin receptors are postulated [107]. Celer et al. showed that the use of teriparatide in patients with postmenopausal osteoporosis increased glucose concentrations, whereas Anastasilakis et al. showed that this trend decreases when teriparatide treatment is continued [108,109]. In summary, there are no randomized trials using these therapies in patients with type 1 diabetes in terms of anabolic osteoporosis therapies. However, as far as type 2 diabetes is concerned, similar increases in BMD and reductions in fracture risk have been shown in T2DM patients as in non-diabetic individuals, and even the increase in BMD of the femoral neck was significantly greater in diabetic patients compared to controls [110].

All in all, anabolic drugs which stimulate both bone resorption and bone formation contribute to bone mass gain through high bone turnover and activation of the Wnt signaling pathway [111,112,113]. This may be particularly effective in diabetic patients with impaired osteoblast differentiation and maturation along with reduced bone formation.

### 4.3. Drugs with a Mixed Mechanism of Action

Drugs with a mixed anabolic-antiresorptive mechanism of action include strontium ranelate. The effect of this substance depends on various mechanisms, e.g., activation of calcium receptors located on osteoblasts and osteoclasts, and a positive effect on the OPG/RANKL ratio. Moreover, it has a proven anti-fracture effect on both the vertebrae and the femoral neck, both in women and in men. Nevertheless, due to possible thromboembolic complications and an increased risk of cardiovascular disease, the European Medicines Agency (EMA) has introduced significant restrictions with regard to its use. However, the study by Atteritano et al., including 40 women with postmenopausal osteoporosis, showed no statistically significant changes in fasting glucose concentrations following one year of strontium ranelate therapy [114].

## 5. Summary and Conclusions

As a systemic disease, diabetes requires a multifactorial approach concerning the proposed therapy and dietary treatment. It is crucial to keep in mind the impact of drugs on bone management and consider dietary recommendations in the prevention and treatment of bone disorders in individuals with diabetes. Although it is currently not recommended to screen for osteoporosis, and there are no particular recommendations for the prevention of osteoporosis in people with type diabetes, it is worth paying attention to the amount of calcium and vitamin D consumed and the frequency of physical activity as well as avoiding potential risk factors for osteoporosis such as smoking. In addition, drug selection for a patient with osteoporosis and diabetes is significant not only in view of osteoporosis therapy but also with regard to the impact on the already disturbed carbohydrate metabolism. Bisphosphonates appear to be a recommended first-line drug for osteoporosis therapy in patients with diabetes. In the elderly and patients with impaired renal function, denosumab is the preferred medication. In contrast, anabolic agents should be considered in patients with severe osteoporosis. Consequently, it can be concluded that dietary management constitutes an essential element of treating and preventing bone mineralization disorders in people with diabetes. Scientific studies confirm the beneficial effect of metformin on bone mineral density in individuals with diabetes. Conversely, drugs from the sulfonylurea group (due to the risk of hypoglycemia) and thiazolidinediones (due to the mechanism of action) are not recommended in people with bone mineral disorders and diabetes. The data regarding the effects of new antidiabetic drugs, such as SGLT2 inhibitors and DPP4 inhibitors, are inconclusive. However, GLP1 analogues show a positive effect on bone mineralization disorders. In fact, in terms of the drugs used to treat osteoporosis in patients with diabetes, the effects of bisphosphonates and denosumab remain best understood.

## Figures and Tables

**Table 1:** Effects of antihyperglycemic drugs on the risk of fractures.

Authors	Type of Paper	Results
Hidayat et al. [42]	Meta-analysis of observational study	Metformin was associated with a reduced risk of fracture
Salari-Moghaddam et al. [43]	Meta-analysis	Metformin was inversely associated with the risk of fracture
Starup-Linde et al. [50]	Original paper	Sulfonylureas were associated with hip fracture in DM2 patients
Losada-Grande et al. [52]	A population-based matched cohort study	Insulin was probably associated with a 38% excess fracture risk among patients with DM2
Zhong-Ning et al. [55]	Meta-analysis of randomized clinical trials	Thiazolidinediones were associated with an increased risk of hip fracture among women
Bilezikian et al. [56]	Randomized controlled trial	52-week therapy of rosiglitazone in postmenopausal women with DM2 was associated with a small reduction in BMD of the total hip, femoral neck, and lumbar spine and increased markers of bone turnover
Cheng et al. [61]	Meta-analysis of randomized clinical trials	GLP-1 agonist (liraglutide and lixisenatide) therapy were associated with a reduced risk of bone fractures
Chen et al. [64]	Meta-analysis of randomized clinical trials	DDP-4 inhibitors did not affect the risk of fractures among DM2 women as compared with other antidiabetic drugs or placebo
Wen-Hsuan et al. [67]	Population-based cohort study	DPP-4 inhibitors were associated with a reduced risk of upper limb fractures among DM2 patients
Watts et al. [69]	Clinical trial	Canaglifozin (Inhibitors of the sodium-glucose-2 cotransporter) increased the risk of fractures
Bilezikian et al. [73]	Randomized controlled trial	Canaglifozin (Inhibitors of the sodium-glucose-2 cotransporter) treatment over 104 weeks (dose 100–300 mg) was associated with a decrease in BMD of the total hip, but not of the femoral neck, lumbar spine, and distal forearm)

## Data Availability

Not applicable.
